# Acceptability of an annual tenofovir alafenamide implant for HIV prevention in South African women: findings from the CAPRISA 018 Phase I clinical trial

**DOI:** 10.1002/jia2.26426

**Published:** 2025-02-21

**Authors:** Tanuja N. Gengiah, Craig J. Heck, Lara Lewis, Leila E. Mansoor, Ishana Harkoo, Nqobile Myeni, Marc M. Baum, John A. Moss, James F. Rooney, Catherine Hankins, Bruno Pozzetto, Salim S. Abdool Karim, Quarraisha Abdool Karim

**Affiliations:** ^1^ Centre for the AIDS Programme of Research in South Africa Durban South Africa; ^2^ Department of Epidemiology Columbia University Mailman School of Public Health New York City New York USA; ^3^ Division of Infectious Diseases, Department of Medicine Columbia University Irving Medical Center New York City New York USA; ^4^ Oak Crest Institute of Science Monrovia California USA; ^5^ Gilead Sciences, Inc. Foster City California USA; ^6^ Amsterdam Institute for Global Health and Development Amsterdam The Netherlands; ^7^ Centre International de Recherche en Infectiologie (CIRI), Team GIMAP Jean Monnet University of Saint‐Etienne Saint‐Etienne France

**Keywords:** acceptability, clinical trial, HIV prevention, implant, PrEP, tenofovir alafenamide

## Abstract

**Introduction:**

Long‐acting HIV pre‐exposure prophylaxis promises to improve uptake, adherence and persistence challenges experienced with daily oral tablets. We assessed the acceptability of an annual tenofovir alafenamide (TAF) implant in South African women enrolled from 9 July 2020 until 31 May 2022 in a Phase I trial.

**Methods:**

Six women received one TAF implant for 4 weeks (Group 1), after which 30 women were randomized (4:1, TAF to placebo ratio) to receive 1 or 2 TAF or placebo implants for 48 weeks (Group 2), before trial discontinuation. Acceptability assessments were conducted pre‐ and post‐implant removal. Implant attributes (size, quantity, insertion site, palpability, visibility) and physical experiences (insertion/removal procedures, implant site reactions [ISRs]) were rated on a scale of 1 (highly unacceptable) to 6 (highly acceptable), with 4 being the acceptability threshold. The mean (range) of the mean acceptability scores across all pre‐removal visits were calculated, including stratification by removal timing (early vs. scheduled). Implant likes and dislikes were also assessed.

**Results:**

The median participant age was 26 years. Prior to implant removal, the mean (range) acceptability scores were 5.4 (3.6–6.0) for product attributes and 5.1 (1.7–6.0) for physical experiences. Eleven (31%) participants had early implant removals, occurring on average 19 weeks (range 2–27 weeks) after insertion. The proportion of study visits reporting adherence measure as unacceptable in early versus scheduled removals: ISRs (50% vs. 19%), visibility (30% vs. 15%), palpability (14% vs. 8%), pain (16% vs. 4%) and implant quantity (13% vs. 1%). Pre‐removal acceptability scores for ISRs (*p* = 0.003) and physical experiences (*p* = 0.05) were significantly associated with early removal. Overall, mean (range) acceptability scores were 5.8 (4.0–6.0) and 5.9 (4.7–6.0) for lifestyle compatibility and likelihood of recommendation, respectively. After removal, 39% of participants found ISRs unacceptable, followed by 22% citing implant visibility. Potential for long‐term HIV protection, followed by discreet and convenient use, were most liked, while ISRs were the most disliked aspect.

**Conclusions:**

While implant attributes, physical experiences and insertion/removal procedures were largely acceptable, local ISRs significantly reduced tolerability and acceptability, resulting in higher‐than‐expected early removals. The potential benefits of an annual TAF implant may be undermined unless tolerability is improved.

## 1 INTRODUCTION

Globally, 44% of new HIV acquisitions in 2023 occurred among women and girls, and within sub‐Saharan Africa, they accounted for 66% of all incident cases [1]. In addition, adolescent girls and young women (AGYW, aged 15–24 years) in this region were over three times more likely to contract HIV than their male counterparts, translating to 3100 incident cases occurring weekly [2]. Given their ongoing vulnerability to HIV acquisition, prioritizing effective prevention strategies for AGYW remains imperative [3].

While daily oral pre‐exposure prophylaxis (PrEP) is highly effective in preventing HIV, inconsistent adherence is the main barrier to effective use in AGYW [4, 5, 6], while PrEP stigma, knowledge gaps and misconceptions reduce acceptability [4]. Furthermore, preferences among PrEP users may evolve over time to better suit their needs. As a result, there is a growing interest in the development of long‐acting (LA) and multipurpose technologies within the HIV prevention and sexual and reproductive health field [7]. LA antiretroviral (ARV) drugs are powerful HIV prevention tools for women. Unlike daily oral pills, they provide extended dosing intervals and enable discreet use. They offer women PrEP choices that may better align with their preferences while addressing the challenges of poor adherence and persistence [8].

Cabotegravir LA injectable (CAB‐LA) has been shown to be highly acceptable, with preference for CAB‐LA injectable over other prevention methods being demonstrated among both men and women in Phase II CAB‐LA trials [9, 10]. Similarly, the Phase III HPTN 084 study involving 3224 African women reported high tolerability and low discontinuation rates (<2%) [11]. The recent successes with the highly effective 6‐monthly lenacapavir injections are promising for the field and data on acceptability is eagerly awaited [12]. The acceptability of the monthly dapivirine vaginal ring in clinical trials was also reported to be high, primarily due to its ease of use and comfort, with 97% of trial users expressing interest in future use [13, 14]. These findings support the suitability of LA formulations for women in particular.

With ARV‐containing sub‐dermal implants for HIV prevention, acceptability assessments have focused on intended use among end‐users: highly favoured attributes include dosing at intervals of greater than 6 months, no interference with sex, minimal adherence challenges and discreet usage [15, 16, 17]. Providers’ perspectives supporting overall acceptability over oral PrEP were high, but there were reservations about the time needed for the implantation procedure and concerns about breakage during removal [18]. Thus far, only islatravir and tenofovir alafenamide (TAF) ARV implants have been tested in clinical trials [19, 20, 21]. While the two islatravir implant trials reported reasonable tolerability after 12 weeks of use, implant acceptability has not yet been reported [19, 21].

In this study, we report the acceptability of an annual sub‐dermal implant among women participating in the CAPRISA 018 first‐in‐human Phase I TAF implant trial.

## 2 METHODS

### 2.1 Study design

The aim of the CAPRISA 018 Phase I/II trial was to evaluate the safety, acceptability, tolerability and pharmacokinetics of sub‐dermally inserted TAF implants, as a potential annual HIV prevention method, in women assessed to be at low risk for HIV. An 8‐item HIV risk assessment tool, allowing binary responses, was used to screen participants to determine eligibility for further health screening and enrolment [20]. Participants were classified as low risk if they had one or no sex partners, no current or recent (past 6 months) sexually transmitted infection (STI), did not practice anal sex, consistently used condoms, did not engage in transactional sex, had a partner less than 5 years older and knew their partner to be HIV negative and monogamous. The Phase I trial was conducted at an urban research clinic in Durban, South Africa, from 9 July 2020 to 31 May 2022.

Consenting, healthy women aged 18–40 years of age were enrolled. Briefly, the Phase I trial included a 4‐week open‐label, first‐in‐human *in‐situ* evaluation of 110 mg TAF silicone implants, 40 mm in length where six women were enrolled sequentially (Group 1). Following Data Safety Monitoring Board (DSMB) review of Group 1 safety, Group 2 was enrolled in a 48‐week double‐blinded, randomized assessment with dose escalation. Thirty women were randomized to receive either one or two TAF or placebo implants in a 4:1 active to placebo ratio. Safety, tolerability and pharmacokinetic outcomes are reported elsewhere [22]. In consultation with the study DSMB, we chose not to proceed with enrolling participants into Group 3 (*n* = 24) to test further dose escalation or Group 4 (Phase II extended safety trial, *n* = 490) due to the low tolerability observed when 11 participants from Group 2 had implants removed earlier than scheduled.

### 2.2 Measurement of acceptability

Self‐reported acceptability data were collected by trained study staff administering structured questionnaires at baseline and at follow‐up visits. Group 1 participants received a single TAF (110 mg) implant inserted for 28 days, with acceptability assessments performed at pre‐removal days 0 (*implant insertion*), 1, 7, 14, 28 (*implant removed*) and post‐removal days 35, 42, 56 (*study exit*) visits. In Group 2, pre‐removal acceptability data were collected at weeks 0 (*implant insertion*), 1, 4, 8, 12, 24, 36, 48 (*implant removed*) and post‐removal acceptability at week 52 (*study exit*). Among women who had early implant removal (prior to the scheduled removal date), post‐removal acceptability was assessed 4 weeks after implant removal.

**Figure 1:**
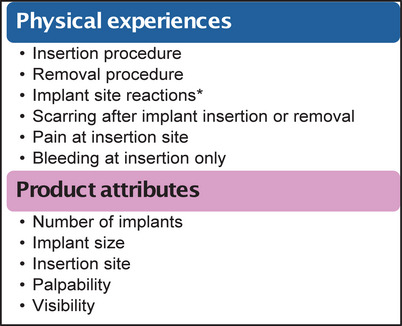
Defining implant acceptability by physical experience and product attribute domains. *Implant site reactions (ISRs) are any side effect experienced at the insertion site. * Any side effect at the insertion site.

Depending on the group assignment, either one or two implants were inserted in the subcutaneous space of the upper non‐dominant arm in the bicep region. Implant acceptability evaluations were analysed across two domains: *Physical experiences* (blue) and *Product attributes* (pink)—the components of each are presented in Figure 1.

Among physical experiences, implant site reactions (ISRs) refer to any side effects at the implant insertion site. Furthermore, bleeding (evaluated at insertion only), scarring and pain are ISRs of special interest, and their acceptability was assessed individually.

Other implant acceptability measures evaluated included the likelihood of peer recommendation, lifestyle compatibility and long‐term HIV prevention potential which were all assessed both before and after implant removal. Participants were asked whether they found the acceptability measure acceptable or unacceptable, then rated the degree of their response as “A little,” “Somewhat” or “A lot.”

Furthermore, we examined pre‐defined implant use *likes* (nothing, ease and convenience, potential long‐term HIV protection, discreetness, assistance with adherence from provider insertion/removal) and *dislikes* (nothing, painful insertion/post‐insertion, clinic visit required for insertion/removal, partner dissatisfaction, post‐insertion bleeding, side effects, visibility to others, anxiety about additional implants, anxiety about the removal process). Participants could report multiple likes or dislikes, and an open‐ended “other” option was made available for additional self‐reported responses.

### 2.3 Statistical analysis

Percentages and frequencies were reported for categorical variables and medians and interquartile ranges (IQR) for continuous variables.

Acceptability scores ranged from 1 to 6. Unacceptable measures were scored as 1 = “A lot,” 2 = “Somewhat” and 3 = “A little.” Acceptable measures were scored as 4 = “A little,” 5 = “Somewhat” and 6 = “A lot.” The percentage of participants reporting that an aspect of the implant was unacceptable (score ≤ 3) was calculated at each visit, using responses from all participants who completed the acceptability interview at that visit. The mean and range of mean acceptability scores across all pre‐removal visits were calculated for each of the acceptability measures. A threshold of 4 was used to indicate overall acceptability.

Fisher's exact tests (categorical variables) and Wilcoxon rank sum tests (continuous variables) were used to compare the baseline characteristics, follow‐up characteristics, and mean pre‐removal scores of those with and without early removals.

Finally, we calculated the percentage of participants who liked or disliked an implant characteristic, first by averaging their binary (yes/no) responses for a given characteristic across visits to obtain a mean value per participant, and then averaging these mean values to obtain an overall mean value. Where relevant, results were stratified by study group, number of implants, TAF or placebo condition and early versus scheduled removals.

All statistical analyses were conducted in SAS version 9·4 (SAS Institute Inc., Cary, NC, USA).

### 2.4 Regulatory and ethics approval

Written informed consent was obtained before any trial procedures commenced. The trial was registered on the Pan African Clinical Trials Registry#: PACTR201809520959443 and SANCTR#. Regulatory approvals were obtained from the University of KwaZulu‐Natal Biomedical Research Ethics Committee (BFC107/18) and the South African Health Products Regulatory Authority (reference: 2018052).

## 3 RESULTS

Overall, 36 participants met eligibility criteria and were enrolled in the trial: six in Group 1 and 30 in Group 2. At baseline (Table 1), the median age was 26 years across groups (Group 1 IQR: 20.5–33.8 years, Group 2 IQR: 23.0–29.0 years). Thirty participants received TAF implant/s and six had placebo implant/s inserted. Contraceptive implant use experience was reported in 14% of study participants.

**Table 1:** Baseline participant characteristics

Variables	All participants (*N* = 36)
Age in years, median (IQR)	26 (22–30)
Body mass index, median (IQR)	28 (25–32)
Mid‐upper arm circumference in cm, median (IQR)	30 (27–33)
Education: completed Grade 12 or above, *n* (%)	26 (72%)
Employment status: unemployed, *n* (%)	34 (94%)
Race: Black African, *n* (%)	36 (100%)
Previous contraceptive implant use history, *n* (%)	5 (14%)
^a^Relationship status: in a non‐casual relationship, *n* (%)	33 (92%)
Group 1 *n* (%)	
1 TAF 110 mg implant	6 (100%)
Group 2 *n* (%)	
1 TAF 110 mg implant	12 (40%)
1 Placebo implant	3 (10%)
2 TAF 110 mg implants	12 (40%)
2 Placebo implants	3 (10%)

Abbreviations: IQR, interquartile range; TAF, tenofovir alafenamide.

^a^ Eight percent reported no sex partner at baseline.

**Figure 2:**
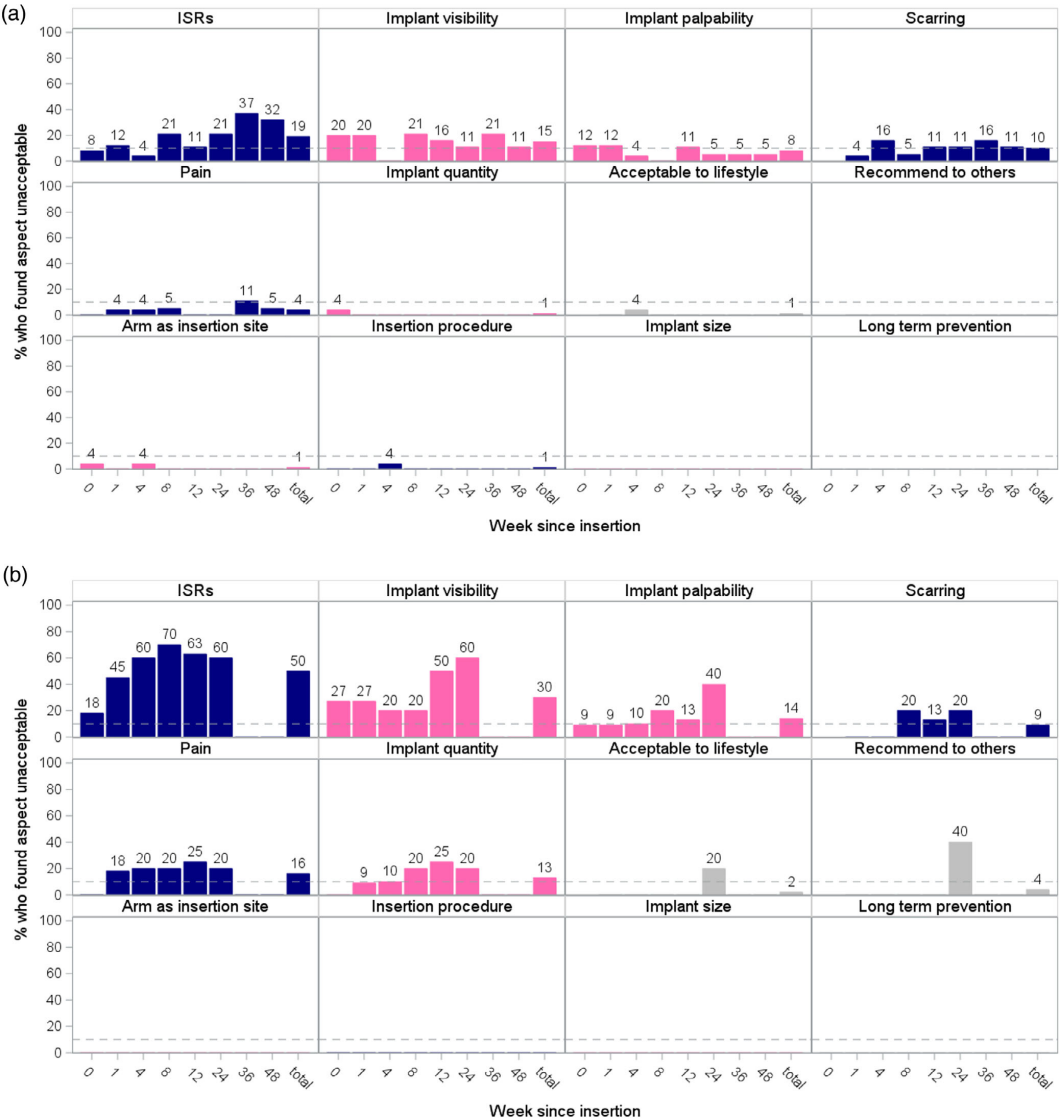
Percentage of participants reporting an implant characteristic as being unacceptable, by study visit and by scheduled (a) and early (b) removal. (Dashed line represents 10% acceptability threshold). Physical experiences denoted by blue bars, product attributes denoted by pink bars and grey bars represent other acceptability measures. ISR, implant site reaction.

The percentage of participants reporting that an aspect of the implant was unacceptable at a study visit is shown for each of the acceptability measures by removal timing (scheduled or early) (Figure 2). These data are represented up until the point at which their implant was removed.

### 3.1 Implant site reactions and acceptability

Overall, ISRs were reported as being unacceptable at 26% of all study visits, implant visibility at 19%, scarring at 10% and implant palpability at 9% of all visits. Among participants who had early implant removal, ISRs were reported as being unacceptable at 50% of all visits, implant visibility at 30%, palpability at 14%, scarring at 9%, pain at 16% and implant quantity at 13%. Among participants who removed their implants on schedule, ISRs were reported as being unacceptable at 19% of all visits, implant visibility at 15%, palpability at 8%, scarring at 10%, pain at 4% and implant quantity at 1%.

Overall mean (range) pre‐removal acceptability scores are represented in Table 2; mean scores overall and by sub‐group were at or above the acceptability threshold of 4 for all acceptability measures except for ISRs.

**Table 2:** Mean (minimum–maximum range) of participant mean pre‐removal acceptability scores by group and implant type

		Group 1	Group 2			
Measure	All	One TAF implant	One TAF implant	One placebo implant	Two TAF implants	Two placebo implants
*N*	36	6	12	3	12	3
**Product attributes**	5.4 (4.3–6)	5.1 (4.3–5.7)	5.6 (4.6–6)	5.4 (4.4–5.9)	5.3 (4.6–6.0)	5.8 (5.6–6.0)
Implant size	5.8 (5.0–6.0)	5.8 (5–6.0)	5.9 (5.3–6.0)	6.0 (6.0–6.0)	5.7 (5–6.0)	5.7 (5.4–6.0)
Implant quantity	5.6 (3.0–6.0)	5.7 (4.5–6.0)	6.0 (6.0–6.0)	6.0 (6.0–6.0)	5.0 (3–6.0)	5.6 (5.1–6.0)
Arm as insertion site	5.8 (4.5–6.0)	5.5 (4.5–6)	5.9 (5.1–6.0)	6.0 (6.0–6.0)	5.9 (5.3–6.0)	6.0 (6.0–6.0)
Implant visibility	4.7 (1.2–6.0)	4.3 (1.8–5.5)	4.8 (2.7–6.0)	4.3 (1.2–5.9)	4.7 (3.0–6.0)	5.7 (5.1–6.0)
Implant palpability	5.2 (2.3–6.0)	4.5 (2.3–5.5)	5.3 (3.3–6.0)	4.7 (2.6–6.0)	5.3 (4–6.0)	5.9 (5.6–6.0)
Physical experience	5.1 (4.0–6.0)	5.2 (4–6.0)	5.0 (4–6.0)	4.9 (4.4–5.3)	5.3 (4.6–6.0)	5.5 (5–6.0)
Insertion procedure	5.6 (4.0–6.0)	5.4 (4–6.0)	5.4 (4.5–6.0)	5.8 (5.6–6.0)	5.8 (4–6.0)	6.0 (5.9–6.0)
Insertion site pain	5.4 (3.0–6.0)	5.4 (3–6.0)	5.1 (3.3–6.0)	5.5 (4.6–6.0)	5.6 (4.4–6.0)	5.6 (4.7–6)
Any ISR	4.3 (1.0–6.0)	4.3 (2.3–6.0)	4.2 (2.3–6.0)	3.4 (1–5.4.0)	4.3 (2.7–6.0)	5.1 (4.6–6)
Scarring	5.2 (3.5–6.0)	5.6 (4.7–6.0)	5.1 (3.5–6.0)	4.6 (4.2–5.0)	5.4 (4.5–6.0)	5.3 (4.8–6)
Insertion bleeding	6.0 (6.0–6.0)	6.0 (6.0–6.0)	6.0 (6.0–6.0)	6.0 (6.0–6.0)	6.0 (6–6.0)	6.0 (6.0–6.0)
**Other measures**						
Acceptable to lifestyle	5.8 (4.0–6.0)	5.5 (4.0–6.0)	6.0 (5.8–6.0)	5.9 (5.8–6.0)	5.7 (4–6.0)	5.9 (5.8–6.0)
Recommend to others	5.9 (4.7–6.0)	5.8 (5.3–6.0)	6.0 (5.8–6.0)	5.9 (5.8–6.0)	5.8 (4.7–6.0)	5.9 (5.8–6.0)
Long‐term prevention	5.9 (5.5–6.0)	5.9 (5.5–6.0)	6.0 (5.7–6.0)	6.0 (6.0–6.0)	5.9 (5.6–6.0)	6.0 (5.9–6.0)

### 3.2 Removal timing and acceptability

There were no unscheduled removals in Group 1. In Group 2, 37% (11/30) of participants had their implants removed early. These comprised five participants with one implant (one placebo, four TAF) and six participants with two implants (six TAF). Six women requested that the implant be removed due to ISRs and the other five were clinician‐initiated removal (four due to ISRs and one due to participant relocation to a neighbouring country). The median time until early removal was 19 weeks (range: 2–27 weeks). Nineteen women from Group 2 had the implant/s inserted for the full 48‐week follow‐up period.

An exploration of the association (Table 3) between baseline characteristics, follow‐up characteristics and pre‐removal acceptability scores with an indicator for early removal showed that the pre‐removal scores for local side effects were strongly associated with early removal.

**Table 3:** Association between participant characteristics and acceptability scores accounting for implant removal timing (early vs. scheduled)

Variables	Early removals median (IQR) or *n* (%)	Scheduled removals median (IQR) or *n* (%)	*p*‐value
*N*	11	25	–
**Baseline characteristics**			
Age in years	28 (21–29)	25 (22–30)	0.824
Body mass index	27.1 (24.2–32.4)	28.6 (25.1–31.2)	0.563
Mid‐upper arm circumference in cm	32.5 (26.8–34.6)	30 (27–32)	0.574
Education: completed Grade 12 or above	7 (64%)	19 (76%)	0.689
Employment status: unemployed	9 (82%)	25 (100%)	0.087
Previous contraceptive implant use	2 (18%)	3 (12%)	0.631
Relationship status: in a relationship	10 (91%)	23 (92%)	1.000
Active TAF implant: yes	10 (91%)	20 (80%)	0.643
Number of implants: two implants	6 (55%)	9 (36%)	0.465
Follow‐up pre‐removal data			
Experienced a moderate (Grade 2) or severe (Grade 3) ISR	8 (73%)	11 (44%)	0.156
Acquired a new sex partner	1 (9%)	1 (4%)	0.524
**Mean (min–max range) pre‐removal acceptability score**			
Product attributes	5.4 (4.7–5.5)	5.7 (5.2–5.9)	0.075
Size	6.0 (5.4–6.0)	6.0 (5.7–6.0)	0.761
Quantity	6.0 (5.0–6.0)	6.0 (5.7–6.0)	0.579
Arm as insertion site	6.0 (6.0–6.0)	6.0 (5.9–6.0)	0.304
Visibility	4.5 (3.2–5.2)	5.3 (4.7–5.7)	0.059
Palpability	5.0 (4.7–5.8)	5.6 (5.0–5.9)	0.299
Physical experiences	5.0 (4.4–5.3)	5.5 (4.9–5.6)	0.047^*^
Insertion procedure	5.8 (5.5–6.0)	5.8 (5.4–6.0)	0.656
Pain	6.0 (4.4–6.0)	5.9 (5.3–6.0)	0.972
Implant site reactions	3.3 (2.7–4.0)	5.0 (4–5.4.0)	0.003^*^
Scarring	5.5 (4.8–6.0)	5.3 (4.8–6.0)	0.793
Bleeding at implant insertion	6.0 (6.0–6.0)	6.0 (6.0–6.0)	1.000
**Other acceptability measures**			
Lifestyle acceptability	6.0 (6.0–6.0)	6.0 (5.8–6.0)	0.807
Recommended to others	6.0 (6.0–6.0)	6.0 (6.0–6.0)	0.719
Long‐term HIV prevention	6.0 (6.0–6.0)	6.0 (5.9–6.0)	0.532

Abbreviations: ISRs, implant site reactions; max, maximum; min, minimum; TAF, tenofovir alafenamide.

* Statistically significant.

Mean pre‐removal acceptability scores for side effects among those with early removal were lower than among participants with scheduled removals. Moderate and/or severe ISRs occurred more frequently among participants with early removals (73%) than among participants with scheduled removals (44%).

Most participants (Group 1: 83%, Group 2: 100%) liked something about the implants, with the possibility of long‐term HIV prevention being the most common (Group 1: 50%, Group 2: 73%) followed by discreetness (Group 1: 33%, Group 2: 33%).

While implant dislikes were less frequent (Group 1: 50%, Group 2: 57%), participants were most concerned about potential ISRs (Group 1: 33%, Group 2: 43%).

**Figure 3:**
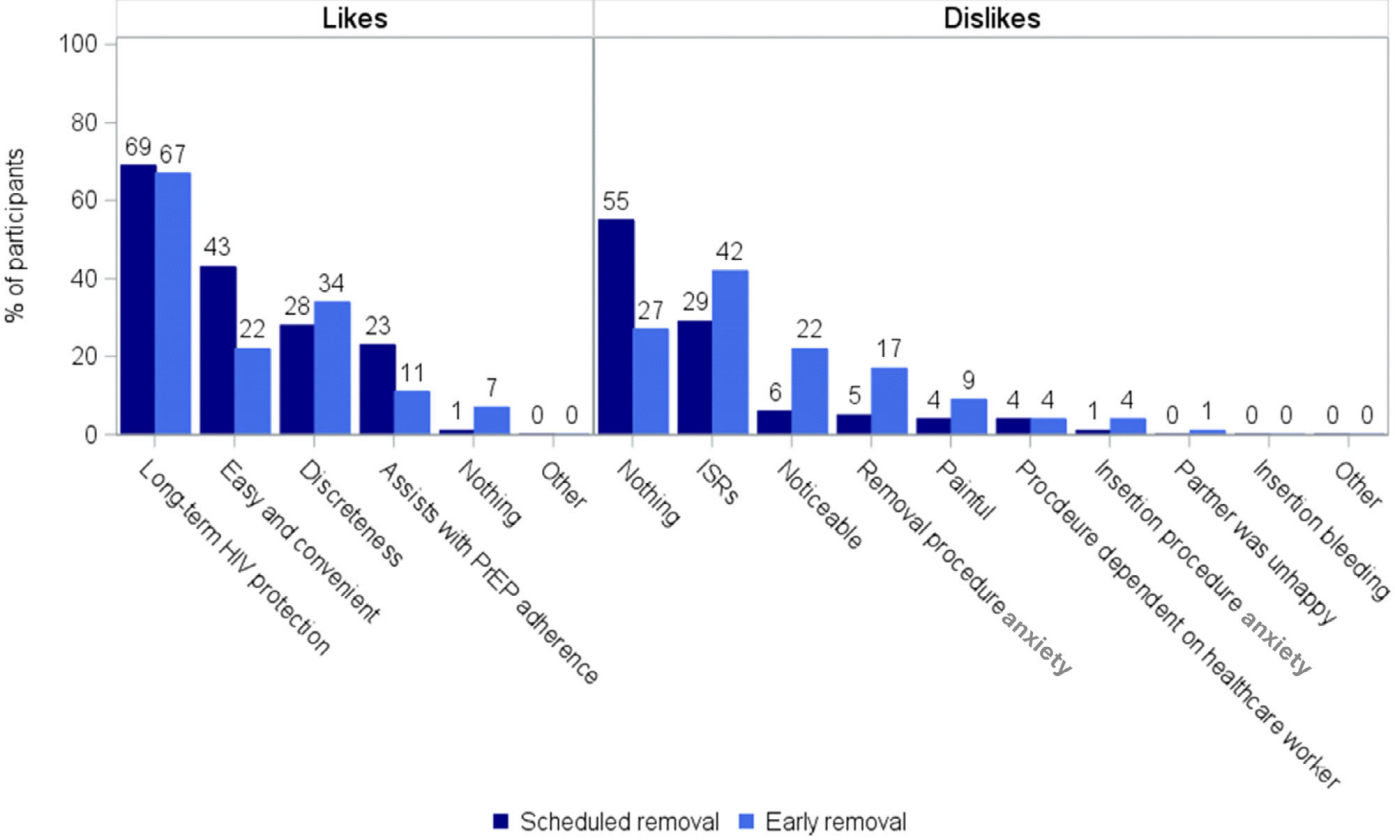
Implant likes and dislikes among scheduled and early removers. ISRs, implant site reactions.

When likes and dislikes were categorized by removal timing (early vs. scheduled) and implant type (active or placebo) (Figure 3), dislikes include ISRs (42%vs. 29%), others may notice (22% vs. 6%), nothing (21% vs. 52%) and removal procedure anxiety (17% vs. 5%), respectively.

### 3.3 Post‐removal implant acceptability

**Figure 4:**
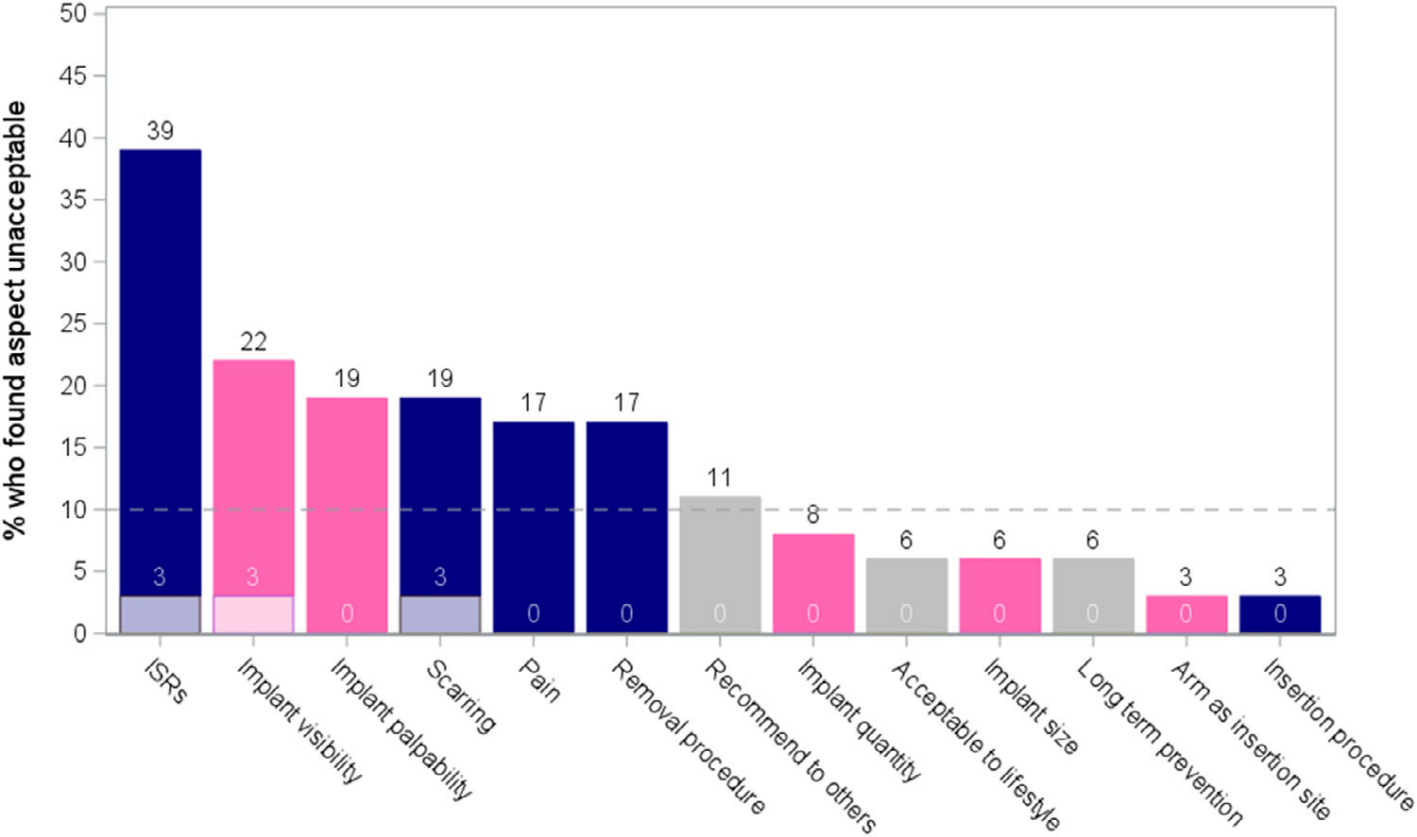
Percentage of participants reporting an implant aspect as unacceptable after implant removal. Physical experiences denoted by blue bars, product attributes denoted by pink bars and grey bars represent other acceptability measures. Shaded portion of bar represents contribution of placebo implant recipients. ISRs, implant site reactions.

After implant removal (Figure 4), ISRs were found to be unacceptable by 39% of participants, followed by implant visibility (22%). Among placebo implant recipients, 3% found ISRs, implant visibility and scarring to be unacceptable, with the remaining concerns were attributed by TAF implant recipients.

## 4 DISCUSSION

An annual implant for HIV prevention could present several advantages to women wanting to reduce their HIV risk but have difficulty with adhering to daily oral PrEP, discreet vaginal ring use or are not accepting of LA injections. This analysis assessed the acceptability of an annual TAF implant in healthy women at low HIV risk enrolled in a Phase I, first‐in‐human trial. Despite low tolerability, with over a third of women having the implant/s removed early, the TAF implant demonstrated high overall acceptability across measures. Notably, earlier‐than‐scheduled implant removals were significantly associated with lower acceptability scores, both for physical experiences as an aggregate measure and for ISRs as an individual variable. Among women who reported the removal procedure experience to be unacceptable, all had received TAF implants. The potential for long‐term HIV protection was the most liked aspect regardless of whether the implant was removed on schedule or earlier than planned.

Several cross‐sectional studies assess the prospective or hypothetical acceptability of sub‐dermal implants for HIV prevention [16, 17, 23] or as a multipurpose technology (MPT) including HIV, pregnancy or STI prevention indications in women [24, 25]. This study is the first to report first‐hand ARV implant user experiences and acceptability outcome measures. Overall, all participants found the product attributes: implant size, quantity of implants (1 or 2) and the arm as the insertion site to be above the acceptability threshold score. These physical properties of the TAF implant, modelled after the contraceptive implant, were well‐received by study participants, an important consideration for future implant development.

In this study, implant tolerability was low and the 11 early removals during the 48‐week follow‐up period were higher than anticipated with significant implications for the future development of the TAF implant technology. Young African women, when considering implants for HIV prevention, have indicated a strong preference for potential implants for discreet use, with minimal visibility under the skin to mitigate concerns with stigma [16, 17, 24] and have minimal side effects [25]. Overall, ISRs were reported unacceptable at 26% of all study visits and were significantly associated with early removal. Furthermore, those with early removal also had a higher proportion of moderate to severe ISRs, contributing to the lower tolerability observed. ISRs resulted in greater implant visibility, and this was reported unacceptable at 30% of study visits among early removal participants. Despite this, scarring as a result of implant use scored well above the acceptability threshold overall and also among early removals. This study reaffirms the critical need to align the technology with previously reported user preferences, as the consequences were significant, and in conducting this study, we were able to obtain these important insights.

Comparatively with PrEP injections in the HPTN 077 Phase II study of CAB‐LA, more than 75% of participants rated the injection frequency, location and duration as highly acceptable; however, an inverse relationship was found between participants’ reported acceptability of injection site pain and discontinuation [9]. Similarly, in HPTN076, where CAB‐LA injection attributes remained highly acceptable over time [10], African participants demonstrated a higher pain tolerability than United States participants [9, 10]. Likewise, pain acceptability in our Phase I study was high for insertion and removal procedures. In the Phase III trial (HPTN 084) of CAB‐LA in African women, high tolerability and low discontinuation rates (<2%) were also observed. Despite 38% of recipients in the CAB‐LA arm reporting injection site reactions, with pain being the most common symptom, no study product was prematurely discontinued due to these side effects [11]. In contrast, with the TAF implant, we found pain to be an acceptable aspect of implant use, but the ISRs were not found to be tolerable in this cohort of women, as indicated by 31% having early removals. CAB LA acceptability studies [9, 10, 11] suggest that participants in high HIV prevalence settings or at risk for HIV may tolerate discomfort for potential PrEP protection. Unlike injectable PrEP, implants offer reversible use, allowing removal if ISRs become intolerable.

The CAPRISA 018 trial is the first to test the TAF implant in humans for 48 weeks [22] but not the first to test an ARV‐based implant: the islatravir sub‐dermal implant was tested for safety, dosing and tolerability over 12 weeks [19, 21]. Acceptability was not reported for the two completed islatravir trials. While the researchers reported mild to moderate ISRs, there were no premature discontinuations over the scheduled implantation period. However, the short trial duration may have precluded observation of premature removal given that, in our trial, the median time to early removal was 19 weeks.

Studies with providers and potential implant users have expressed concerns about the insertion and removal procedures required for PrEP or MPT implant use, based either on previous negative experiences with contraceptive implant rollouts [26], or concerns with potential pain [16] which may potentially be mitigated by using biodegradable implants [24]. In our experience, 17% of participants found removals and 3% found insertions to be unacceptable with few participants being averse to future implant insertions, even among those who had their implant removed early. Pain, bleeding or consequent scarring at the insertion site did not contribute to lowering tolerability and was highly acceptable.

Among young Southern Africans who were interviewed to assess their future interest in using implants for HIV prevention, most preferred a biodegradable, flexible implant that would minimize palpability and increase discreetness and comfort [17, 24]. However, in our cohort, we found few participants who disliked palpability or clinic attendance for removal procedures. Despite the low tolerability, overall suitability to their own or others’ lifestyle, and potential as a long‐term HIV prevention method was found to be highly acceptable. The majority of the participants found individual aspects of the implants that they liked and only 17% of those who had the implant removed early would not recommend the implant to others. While ISRs determined implant usability, additional qualitative research is necessary to improve our understanding of how these aspects contributed to overall acceptability. In our context, the high acceptability we observed—despite ISRs and low tolerability—may be partially explained by the perceived benefits of HIV protection, even among women at lower risk, in a setting with high HIV prevalence.

We showed high post‐removal acceptability across measures, despite the low tolerability in women who had early removals, possibly highlighting the complexity of women's motivations for using LA‐PrEP. It must be acknowledged that the participants in this Phase I trial were at lower risk for HIV acquisition than the intended population who would use this product and may be less motivated to tolerate ISRs. Despite this, their interest and support for this technology is likely driven by the promise of convenient, discreet and long‐term HIV protection, a highly valued aspect. Whether there may be a level of willingness to tolerate side effects, even to a limited extent, if protection from HIV was assured for women at risk for HIV, cannot be informed by this study's outcome.

This analysis has limitations. The small sample size limits the ability to make robust statistical comparisons between groups. Additionally, the high acceptability scores reported by participants, despite the low tolerability of the implant, highlight the limitations inherent in relying solely on self‐reported quantitative data collection and to an extent the limited generalizability of Phase I trial acceptability data in a low HIV risk population. While there may be a tendency to assume potential bias in the form of the white coat effect, incorporating assessments of both product attributes and physical experience domains was intended to reduce this bias and give a more nuanced understanding of acceptability. In this trial, women had the freedom to request implant removal at any time, as evidenced by six of the 11 Group 2 early removals being initiated by participants themselves.

## 5 CONCLUSIONS

This first‐in‐human trial provides useful insights into the acceptability of a novel LA HIV prevention product informative to the development of future implants. While several aspects of the implant use experience—including its attributes, physical experiences, insertion and removal procedures—were highly acceptable, local ISRs significantly reduced implant tolerability leading to higher‐than‐expected rates of early implant removal. The potential benefits of an annual implant may be overshadowed unless local tolerability is assured. Despite the ISRs experienced, participants valued the implant's potential for long‐term HIV protection.

## COMPETING INTERESTS

All authors declare that they have no competing interests.

## AUTHORS’ CONTRIBUTIONS

SSAK and TNG conceived and designed the trial. The trial was overseen by TNG, SSAK, QAK and IH. JK and ZK collected the clinical and safety data, while LEM and NM provided trial operational oversight. MMB and JAM developed the implant and performed the residual drug analysis. CJH and LL analysed the data. SSAK, TNG, CJH, LL, QAK, CH, BP, MMB and JAM interpreted the data. All authors reviewed the final version of this manuscript and consented to publication.

## FUNDING

The trial was funded by the European and Developing Countries Trial Partnership (EDCTP) (Grant No: SRIA2015‐1061), as a project of the EDCTP2 programme supported by Horizon 2020 as well as the South African National Department of Health and the South African Medical Research Council (Project code 96151). In addition, the Department of Science and Innovation and the National Research Foundation contributed funding for this research through the DSI‐NRF Centre of Excellence in HIV Prevention (UID:96354). Cipla Ltd (India) donated some of the drugs studied, while Gilead Sciences, Inc. donated the TAF API used in the implants studied in the CAPRISA 018 trial.

## Data Availability

Study data sets will be made available to investigators whose proposed use of the data has been approved by the CAPRISA Scientific Review Committee. Requests to access the data can be made through the CAPRISA website (www.caprisa.org).
